# Tunable infrared metamaterial-based biosensor for detection of hemoglobin and urine using phase change material

**DOI:** 10.1038/s41598-021-86700-6

**Published:** 2021-03-29

**Authors:** Shobhit K. Patel, Juveriya Parmar, Vishal Sorathiya, Truong Khang Nguyen, Vigneswaran Dhasarathan

**Affiliations:** 1 Department of Electronics and Communication Engineering, Marwadi University, Rajkot, Gujarat 360003 India; 2 Department of Computer Engineering, Marwadi Unversity, Rajkot, Gujarat 360003 India; 3 Department of Physics, Marwadi University, Rajkot, Gujarat India; 4 Department of Information and Communication Technology, Marwadi University, Rajkot, Gujarat India; 5 Division of Computational Physics, Institute for Computational Science, Ton Duc Thang University, Ho Chi Minh City, 700000 Vietnam; 6 Faculty of Electrical and Electronics Engineering, Ton Duc Thang University, Ho Chi Minh City, Vietnam

**Keywords:** Optical sensors, Metamaterials

## Abstract

This paper reports about the outcomes from an investigation carried out on tunable biosensor for detection using infrared in the range of 1.5 µm and 1.65 µm. The biosensor is made of phase change material formed by different alloy combinations, Ge_2_Sb_2_Te_5_ (GST). The nature of GST allows for the material to change phase with changes in temperature, giving the tunable sensing property for biosensing application. Sensor built with amorphous GST (aGST) and crystalline GST (cGST) in different design structures were tested on different concentrations of biomolecules: hemoglobin (10 g/l, 20 g/l, 30 g/l and 40 g/l); and urine (0–1.5 mg/dL, 2.5 mg/dL, 5 mg/dL and 10 mg/dL). The tunable response observed from the tests demonstrates the potential application of the materials in the design of switching and sensing systems.

## Introduction

Biosensors are usually designed following a three-step process: recognition of biomolecules, triggering of signal, and detection. Recognizing the biomolecules is the first and very important step in designing biosensor system. Biosensors are useful for applications in environmental analysis and agricultural management, particularly in detection of infectious diseases in crops, toxics materials, and pollutants. Biosensors are classified into various categories, such as optical biosensor, electrical biosensor, electronic biosensor, etc., of which, optical biosensors are always in great demand due to its optical properties and quick response to nanoparticles^1,2^. Optical biosensors are further classified into two sub-categories, namely label-free and labeled. Label-free mode refers to a system that directly detects the analyte (biomolecule); in label-based mode, the analyte is tagged with a label that will produce a signal, which can be detected by the sensor^3,4^. The advantages of optical biosensors include cost-effectiveness, less time-consuming, shock-free, etc.^5^. As label-free system does not require the use of label as binding agent^6^, the sensor built with this system is relatively easy to use and particularly useful for rapid detection of surface components^7^. On the other hand, the label-based biosensor is relatively costly and complex, especially when detecting large biomolecules^8^.

Surface plasmon resonance (SPR) technique is one of the most common label-free biosensor system. There is a huge demand for SPR due to its feature, which allows for monitoring of changes in the refractive index near the surface of the sensor. SPR technique works are based on two types of configurations, namely Kretschman and otto. The technique allows for measurement of each bound molecule in a form of shift observed at the surface of the sensor^9,10^. Among the advantages of SPR biosensors are the small size, high-resolution detection, remote sensing feature, etc., making them useful for detection of biochemical molecules, particularly in environmental and clinical applications^11^. SPR system interconnects three things namely biochemical components, optical properties, and electronic systems^12^. The system is used for real-time detection of unlabeled biomolecules in a complex structure^13^. Several biosensor designs constructed using different types of resonator and photonics crystal have been reported in previous studies^14–16^. An investigation on the response of biosensor with leaky wave radiating structure made of graphene integrated on Si_3_N_4_ waveguide was previously reported^14^. On the other hand, it is also possible to design an infrared-biosensor using gold split-ring resonators as reported in^15^, which structure is relatively simple. Additionally, an absorber that is polarization-insensitive can also be designed for tunable absorption response with multiple modes of input excitation^16^. Nanocomposite is another material used in biosensor as it confers a cost-effective procedure^17^. Optical biosensors are considered as a primary sensing method in detection of blood samples, which measure the different refractive indices of blood sample with different concentrations of hemoglobin^18^. Lab-on-a-chip biosensor with integrated optical transducers responds more to electromagnetic interference than to electronic components, which solves the Maxwell’s equation and results in good sensitivity^19^. Graphene oxide and reduced graphene oxide can detect bacteria at the micro level; between the two, reduced graphene oxide is more effective as it produces less noises^20^. Application of a guided mode photonic crystal fiber-based biosensor was previously reported in^21^, which is made of graphene and gold.

Similarly, there are other types of biosensor, which have been reportedly used for different applications. One of the examples is the recent development of biosensor with silicon waveguide-based absorber^22^. Several studies have also reported the use of metasurface-based absorber to achieve tunability and reconfigurable responses in biosensor. Refraction at near infrared region was also reported in biosensor using nanodisk metasurface^23^. Previous study also showed the use of silicon photonic biosensor for detection broad spectrum infrared detection of label-free analyte^24^. Recently, there is a surge in demand for biosensor that allows for rapid diagnosis as an alternative to the expensive and time-consuming methods used in the laboratory. Previously, it was reported that rapid disease detection was achieved via a low-cost procedures using electrochemical biosensor^25^. Development of a single-layer biosensor using gold, biomaterial, and silica was proven possible in the past research. The use of the sensor to determine the concentration of hemoglobin in human blood sample was reported previously^26^. One study demonstrated the use of biosensor built using metamaterial and semiconductor that measure refractive index of analyte at far infrared^27^. Metamaterial can also be applied in various applications: as dual tunable absorbers^28^ of different shapes such as chevron^29^ and wrench^30^; chemical sensing^31^; infrared filter^32,33^; tunable polarizer^34^, microelectromechanical devices^35^; and polarization-insensitive devices^36^. Nanoparticle can also be used in development of biosensor for applications such as medical diagnostic devices^37^. Nanoparticle acts as a wide-angle and multi/wide-band absorber in biosensor. The design of infrared biosensor using metamaterial for specific applications based on geometric parameters have been reported, which include temperature^38^, gas^39^, color^40^ and optical^41^ sensor.

Tuning is very important in designing biosensor, and one of the ways to achieve is by using phase change materials such as GST^42^. The use of GST allows for fine-tuning of absorber and sensor in biosensor. GST metasurfaces have been used in making polarization-insensitive absorber^43^. GST has also been proven to improve the performance of plasmonic devices^44^. GST is the most commonly used phase change material that reversibly changes from the amorphous to crystalline phase. The material exhibits different optical and electrical properties in crystalline and amorphous phase, which is useful for applications such as memory storage, sensor, logical device^45^, etc. Tunable phase change material is particularly beneficial for development of biosensor in sensing and switching applications. GST corresponds well with the light which feature is beneficial for development of nanophotonic and nanoplasmonic tools^46^. GST absorbs light perfectly in crystalline phase compared to amorphous^43^. Addition of gold between the metal layer and metal grating improves the tenability of GST-built biosensor, which results in good sensitivity^47^. The dynamic control over optical absorption in the Au-GST structure is a feature that is useful for development of plasmonic switches, modulators, and metasurfaces.

The different shapes of resonance structures such as plasmon metamaterial in cylindrical and cubical form will be discussed in the next section of this paper. Although the range of concentrations of analyte used in the past investigations differs from one structure to another, this discussion focuses only on detection in the range of wavelength between 1.5 µm and 1.6 µm. The design of resonance structures discussed in this paper have been optimized, and the respective absorption responses have been analyzed to assess sensor sensitivity. A comparative analysis is also presented in this paper to identify the behavior of tunable sensor.

## Design and modelling

**Figure 1:**
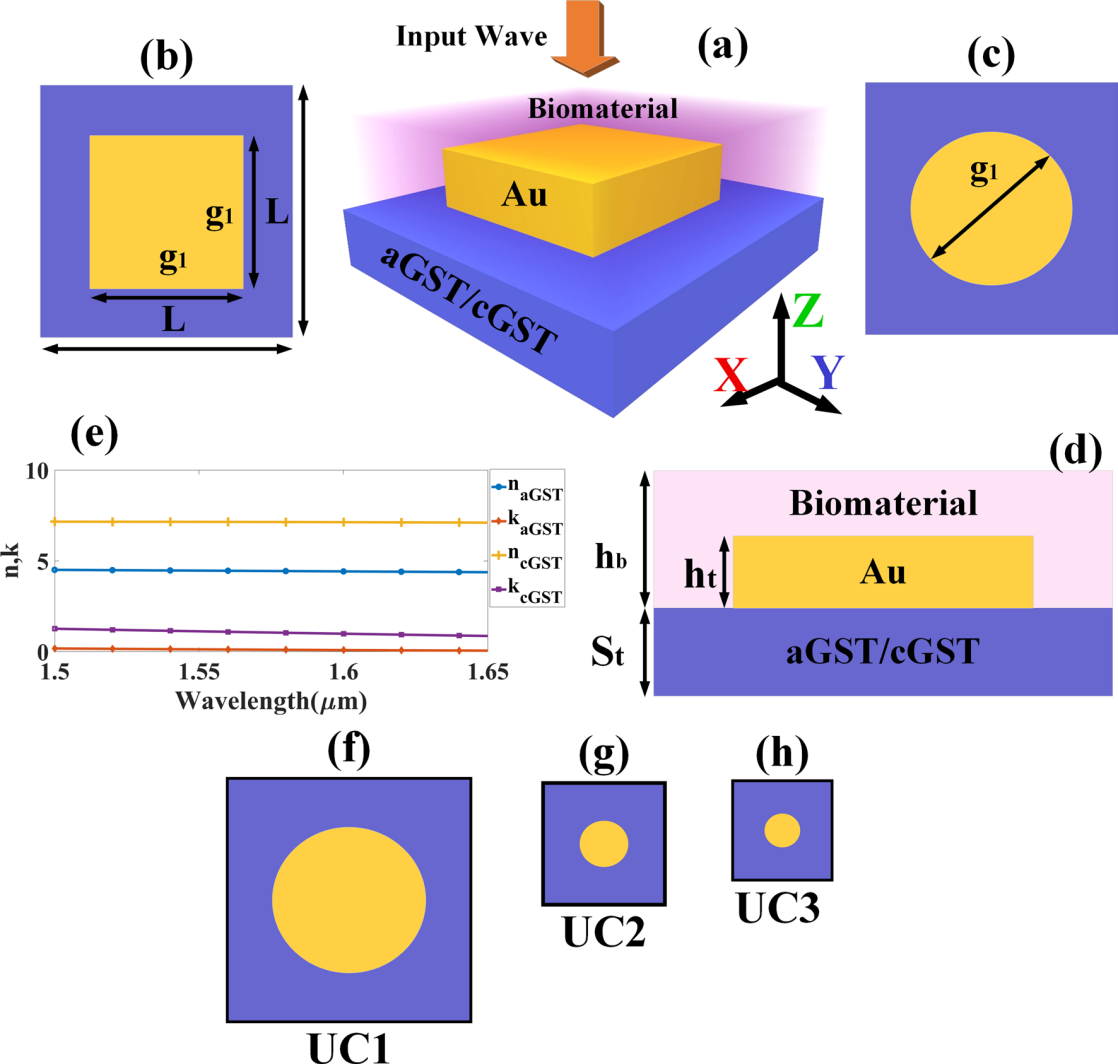
Schematic diagram of the metamaterial cubic and cylinder resonator in the structure of GST-assisted biosensor. (**a**) A 3D view of the sensor. (**b**) Top view of the sensor with metamaterial cubic resonator. (**c**) Top view of the sensor with metamaterial cubic resonator. (**d**) Side view of the sensor. The bottom layer of the structure is made of aGST/cGST. Biomolecule is placed on the top of the sensor. The incident wave is excited along the z-axis. Dimensions of the structure are: *S*_*t*_ = 800 nm, *h*_*t*_ = 600 nm, *h*_*b*_ = 2000 nm, *g*_1_ = 1400 nm, and* L* = 2000 nm*.* (**e**) Real and imaginary refractive index of aGST and cGST within the range of 1.5–1.65 µm. (**f**) Dimension of UC1 is L × L = 2000 × 2000 nm^2^, g1 = 1400. (**g**) Dimension of UC2 is L × L ≈ 666 × 666 nm^2^, g1 ≈ 466. (**h**) Dimension of UC3 is L × L ≈ 400 × 400 nm^2^, g1 ≈ 280.

Figure 1 illustrates the different designs of biosensor incorporated with different shapes of resonance structures, namely cylindrical metamaterial and cubical gold. GST was chosen as the material for the base of the resonance structures, which phase-change characteristics are reflected in the transition of spectral responses via transfer-matrix method. Phase transition of GST from amorphous GST (aGST) to crystalline GST (cGST) was carried out by subjecting the material to pulse energy up to glass transition temperature^48^. Successful applications of phase change materials enables the development of tunable optical sensor, beneficial particularly for artificial intelligence (AI) applications. Array paterns of plasmonic gold in biosensor is proposed in this paper, which are assigned as UC1, UC2 and UC3 to represent the different shapes of resonance structure.

The refractive index of hemoglobin and urine in different concentrations is shown in Table S1 (see Table S1 of supplementary information). The calculated length and width (*L* × *W)* of the structure is 2000 nm. The height of the GST (*S*_*t*_*)* substrate and Gold (*h*_*t*_*)* resonator was determined as 800 nm and 600 nm, respectively. The height *(h*_*b*_*)* of the sensing medium was determined as 2000 nm*.* The proposed design of biosensor was investigated in three structures of unit cell as shown in Fig. 1f–h. The dimensions of the unit cell were assigned as UC1 (Unit Cell 1), UC2 (Unit cell 2), UC3 (Unit cell 3). The dimensions of UC1 was determined as L × L = 2000 × 2000 nm^2^ and g1 = 1400; UC2 with L × L ≈ 666 × 666 nm^2^ and g1 ≈ 466; and UC3 with L × L ≈ 400 × 400 nm^2^ and g1 ≈ 280. For better understanding, numerical investigation of the proposed biosensor was carried out using periodic boundary condition approach for the unit cell. The different absorption peak shifts for the different array of structures are shown in Fig. S1 of supplementary material. Therefore, variation in the number of resonators over the dimension of unit cell grants tunability to both aGST and cGST-based biosensor. The analyte was placed on the top of the gold resonator as shown in Fig. 1a. The incident infrared is emitted from the top and excited along the z-axis. The proposed structure was periodically extended along the x and y-axis. Transverse electric mode (TE) was used to excite the input wave. The structures were numerically investigated using finite element method (FEM). The meshing of the structures was carried using tetrahedral meshing method. Metallic layer was used at the bottom of the proposed structure.

Intensity of absorption (A) depends on parameters such as transmission (T) and reflection (R) by the equation, A = 1 − T − R. A theory was proposed previously^49^, which demonstrates that when the impedance of free space and device is matched, the R = 0; overall transmission will be reduced from $T= {e}^{-2{n}_{2}dk}$ to $T= {e}^{-\alpha d}$: where $k$ is the free space propagation vector, $d$ is the thickness of sample, ${n}_{2}$ is the effective refractive index, and $\alpha$ is the absorption coefficient. Impedance matching by the absorber is very important to achieve maximum absorption. According to the theory of impedance matching, the loss needs to be generated in the space between two conducting materials. The value of ${n}_{2}$ is determined by the refractive index of the spacer material. To achieve near-unity absorption, ${n}_{2}$ must be as large as possible. As shown in Fig. 1e, large infrared refractive index was achieved using biosensor built with aGST and cGST.

## Numerical results

### Absorption spectrum and wavelength shift

**Figure 2:**
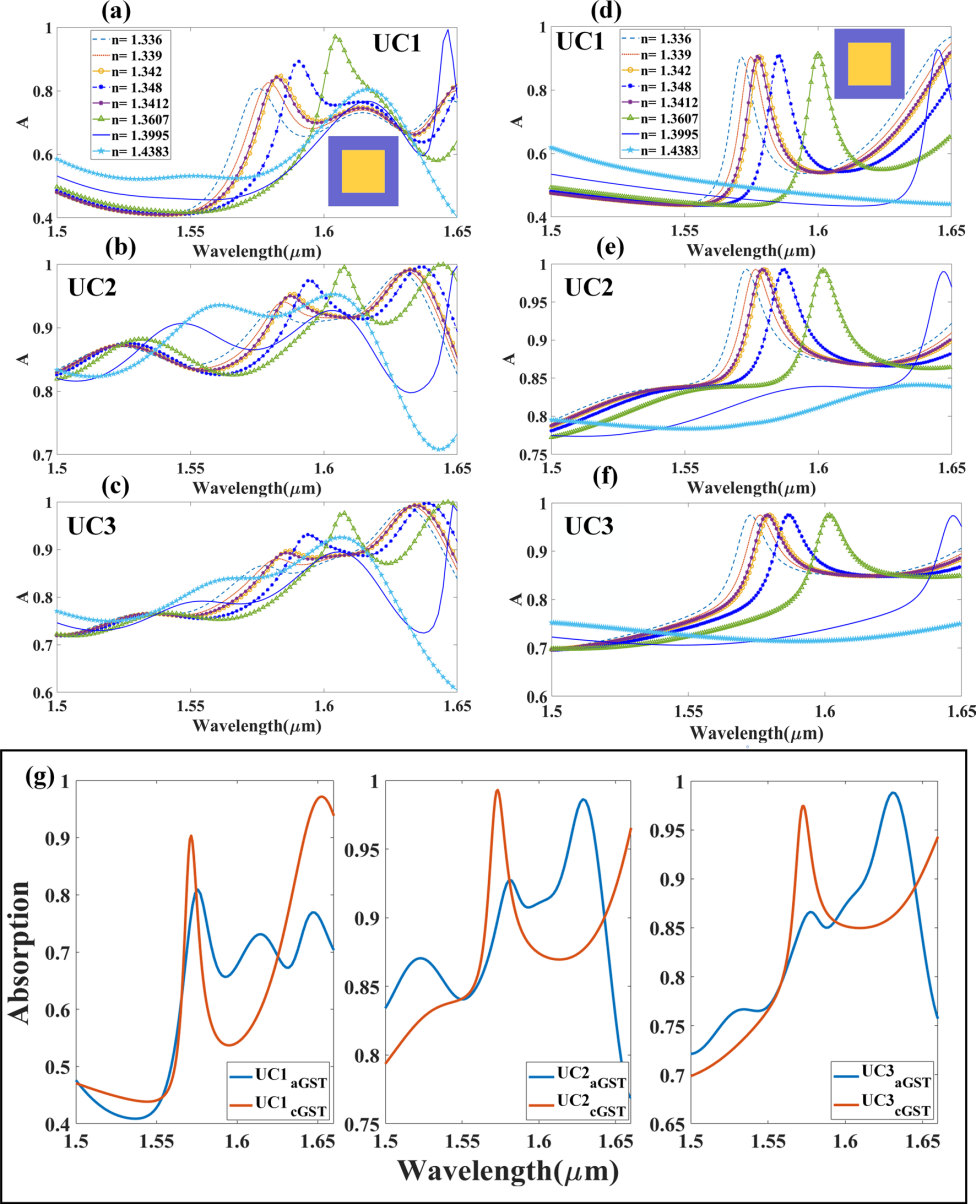
Absorption spectrum of different biomolecules with different refractive indices as detected by biosensor built with metamaterial cubic structure. Absorption spectrum of (**a**) UC1, (**b**) UC2 and (**c**) UC3 metamaterial cubic structure with aGST as substrate. Absorption spectrum of (**d**) UC1, (**e**) UC2 and (**f**) UC3 cubic metamaterial with cGST as substrate. (**g**) Shifting in absorption spectra with different arrays and phases of material.

**Figure 3:**
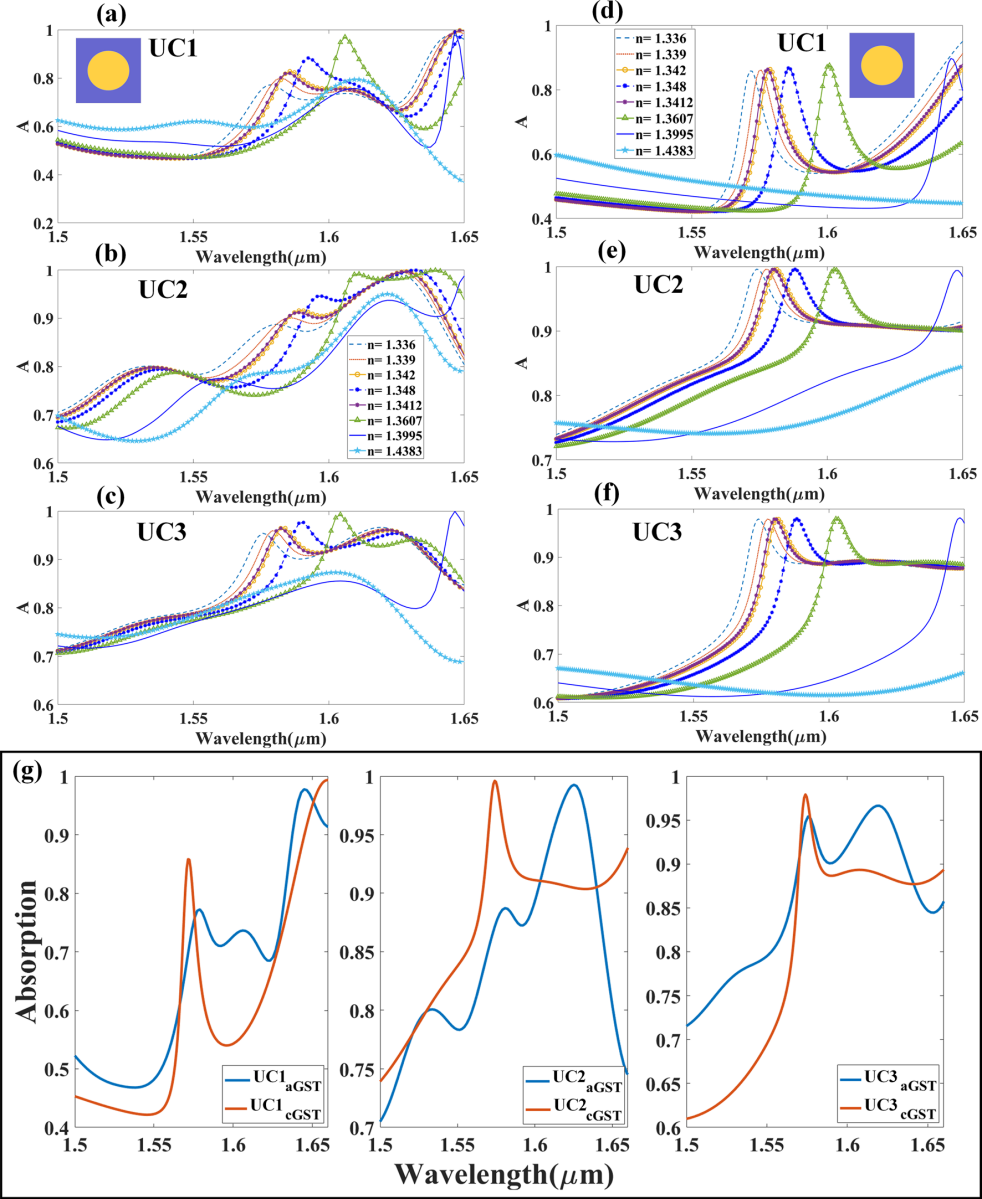
Absorption spectrum for different refractive indices of biomolecules (metamaterial cylindrical structure.). Absorption spectrum of cylindrical metamaterial resonator in (**a**) UC1, (**b**) UC2 and (**c**) UC3 with aGST as substrate. Absorption spectrum of cylindrical metamaterial resonator in (**d**) UC1, (**e**) UC2 and (**f**) UC3 with cGST as substrate. (**g**) Shifting in absorption spectra for different arrays and phases of material.

This section discusses the absorption spectrum of the different arrays of cubical metamaterial resonators constructed with phase change materials, namely aGST and cGST as illustrated in Fig. 2. The absorption spectrum demonstrates the tunable effects of different arrays of resonating structures, with both phases of GST used as substrate. Similarly, Fig. 2d–f shows the variation in intensity of absorption as different array of structures were tested, using cGST in the construction of the biosensor. Variation in the absorption peak of different phases of GST used in cubic resonator arrays is shown in Fig. 2g. The important role of the phase change material is illustrated in Fig. 2g, which shows huge wavelength shift of maximum absorption peak as different arrays of resonator used. As observed in Fig. 2a–f, increase in absorption intensity (peak) is proportional to the increase in refractive indices of analyte. Shift in the wavelength can also be observed in the absorption peak as different concentrations of analyte were used. The wavelength shift as different concentrations of hemoglobin used is lower than that of urine; the difference in the refractive index of different concentrations of hemoglobin is smaller than that of urine. Similarly, Fig. 3 shows the absorption spectrum of the cylindrical metamaterial resonators, with aGST and cGST as substrate, which corresponds to the different refractive indices of the analyte. It is also observed that the wavelength shift similar, while the sensitivity varies as different analytes were used as sample. The results show that the wavelength has shifted for approximately 300 nm as different concentrations of urine were tested, while a shift of about 150 nm can be observed in the wavelength as different concentrations of hemoglobin were tested. The shift in the wavelength as aGST and cGST transition into different phase in cylindrical resonator array is shown in Fig. 3g. Similar to cubic resonator, a huge wavelength shift can also be observed for the cylindrical structure as GST changes phases. The wavelength shifts for both cubic and cylindrical resonators are shown in Fig. S1 (supplementary information); the shifts demonstrate the tunable response of both structures. For aGST-based cylindrical and cubical structures the lowest wavelength of absorption peak is near to 1.57 μm, while the highest wavelength is near 1.6 μm. A shift of 5 nm can be observed near the wavelength of 1.57 μm, and 20 nm shift near to wavelength 1.6 μm for cylindrical and cubical resonators, respectively.

### Wavelength shift for different refractive indices

**Figure 4:**
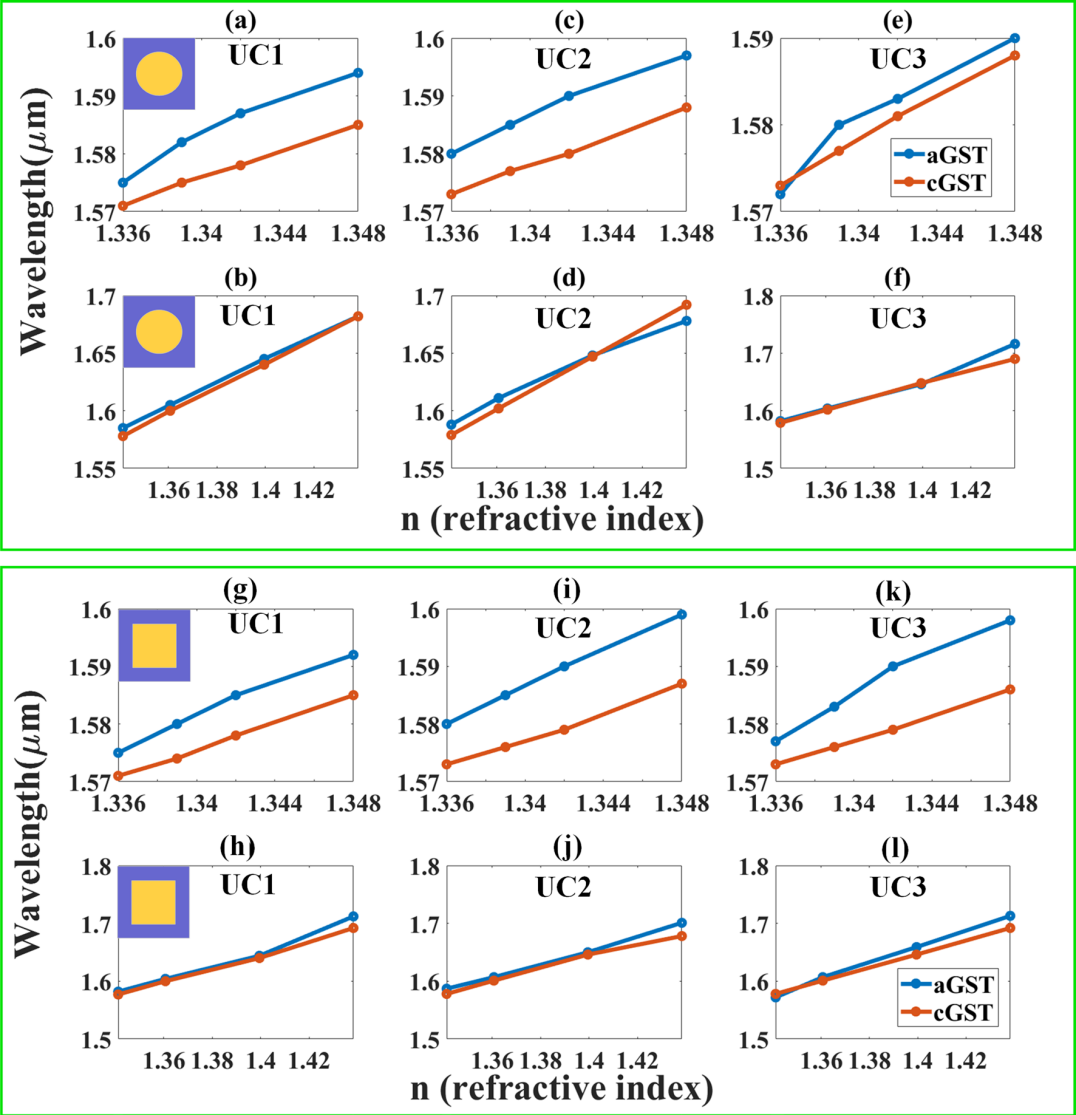
Sensor response (wavelength shift) with *n* (refractive index) for both phases of GST of different metamaterial arrays. (**a**–**f**) Response by cylindrical array-based sensor in UC1, UC2 and UC3. Urine detection is represented in (**a**), (**c**) and (**e**); hemoglobin detection is represented in (**b**), (**d**) and (**f**). (**g**–**l**) Response of cubic array-based sensor in UC1, UC2 and UC3. Urine detection is represented in (**g**), (**i**) and (**f**); hemoglobin detection is represented (**h**), (**j**) and (**l**).

Linear variation in wavelength for different refractive indices resulted from the use of cylindrical resonator array is shown in Fig. 4. Figure 4a, b show the variation in the wavelength shift as biosensor with unit cell of UC1 was used in detection of urine and hemoglobin. Similarly, Fig. 4c, d and Fig. 4e, f show the variation in wavelength shift for biosensor with unit cell of UC2 and UC3, respectively. Figure 4g–l show the variation in wavelength for different refractive indices resulted from the use of cubic resonator array. The shift in wavelength is larger in detection of hemoglobin than urine as observed in Fig, which may be due to the larger variation in refractive indices of hemoglobin. Variation in wavelength indicates the sensitivity of the proposed structure of biosensor. Transitioning of aGST to cGST in biosensor with cylindrical metamaterial resonator resulted in wavelength shift of 26 nm; similarly, a shift of 23 nm in the wavelength can be observed in biosensor with cubic metamaterial resonator. It is important to note that GST is used in biosensor mainly for determining the wavelength shift of the absorption peak as GST changes phases during the operation (i.e., aGST to cGST, or cGST to aGST). In this study, a wavelength spectrum for both aGST and cGST-based biosensors with cylindrical and cubic resonator arrays is proposed based on variations in the wavelength of absorption peak, which takes into account only the clearly exhibited peak as shown in Figs. 2g and Fig. 3g. The variation indicates the tunable response of the biosensor at wavelength in the range of 1.5–1.65 μm.

### Biosensor sensitivity variation

The sensitivity (*ΔS*) is calculated by the ratio of wavelength difference (*Δλ*) and refractive index difference (*Δn*) between two samples of analyte. A comparative analysis over the variations in sensitivity of different structures, biomolecules, and GST phases has been derived (see Table S2 to Table S5 of supplementary information). There are two parameters responsible for variation in sensitivity: (1) refractive index of the analyte; (2) refractive index of material used in biosensor (i.e., aGST and cGST). Variation in sensitivity in detection of different biomolecules can be observed in the proposed designs of biosensor as shown in Fig. 4. The refractive index of the biomolecules plays a key role for impedance-matching of surface resonators. Changes in the refractive index at the top layer of biomolecules may result in changes in resonance point. Therefore, there can be different resonance points over the range of wavelength. The smaller (larger) the difference in refractive index, the smaller (larger) the shift in the wavelength. The sums of variation in wavelength shift ultimately represents the response sensitivity of the structure (i.e., detection of biomolecules in different concentrations). The approximate minimum and maximum sensitivity of cylindrical resonator is 1000 nm/RIU and 2333 nm/RIU, respectively, when tested on urine samples; the structure demonstrates minimum and maximum sensitivity of 825 nm/RIU and 1795 cnm/RIU, respectively, when tested on hemoglobin. On the other hand, the minimum and maximum sensitivity of cubic resonator is 1000 nm/RIU and 2667 nm/RIU, respectively, when tested on urine samples; the structure demonstrates minimum and maximum sensitivity of 773 nm/RIU and 1814 nm/RIU, respectively, when tested on hemoglobin.

### The effects of physical parameters

**Figure 5:**
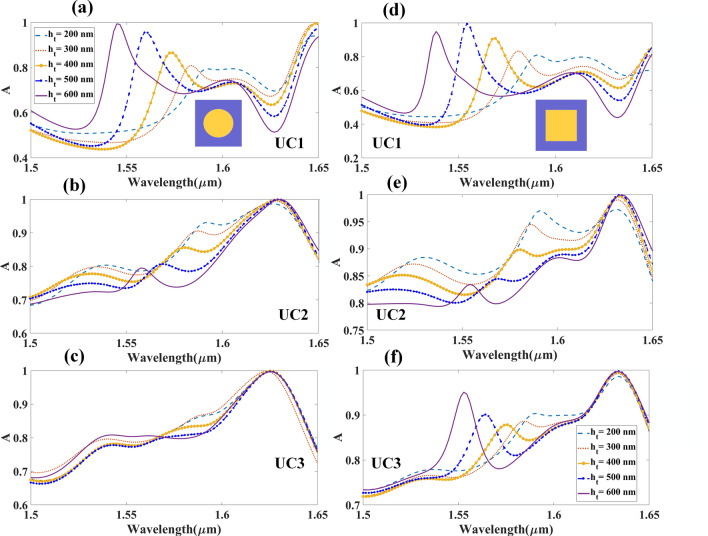
Absorption spectrum of gold resonator with different heights (metamaterial cylindrical and cubical array structures), which varies between 200 and 600 nm. Absorption spectrum for cylindrical metamaterial array with (**a**) UC1, (**b**) UC2 and (**c**) UC3. Absorption spectrum for cubic metamaterial array with (**d**) UC1, (**e**) UC2 and (**f**) UC3.

**Figure 6:**
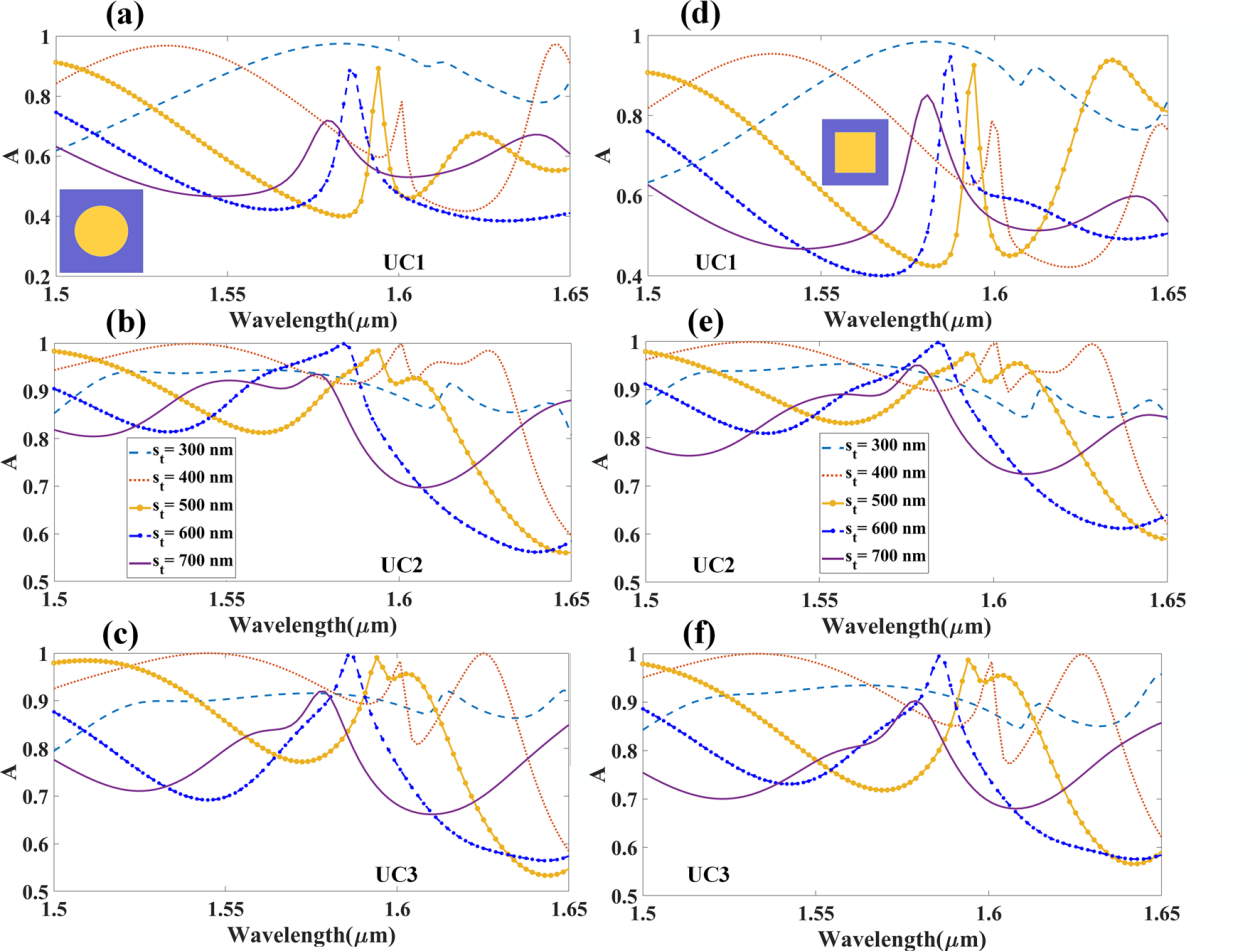
Absorption spectrum for the different heights of aGST substrate (metamaterial cylindrical and cubical array structures), which varies between 200 and 600 nm. Absorption spectrum for cylindrical metamaterial with (**a**) UC1, (**b**) UC2 and (**c**) UC3; absorption spectrum for cubic metamaterial array with (**d**) UC1, (**e**) UC2 and (**f**) UC3.

Variations in absorption spectra have been studied and plotted for different physical parameters as shown in Figs. 5 and Fig. 6. The results are derived from the different *h*_*t*_ ranging from 200 to 600 nm. Figure 5a–c and Fig. 5d–f show the variation in absorption intensity by metamaterial in cylindrical and cubic shapes, respectively. Changing the absorption spectra of the cGST is also possible by varying the physical parameters such as the height of gold and GST substrate.

The absorption spectra of GST in cylindrical and cubic resonator of different heights, *s*_*t,*_ range from 300 to 700 nm are illustrated in Fig. 6. Notable wavelength shift is observed in the absorption peak in unit cell assigned as UC1 as shown in Figs. 5 and Fig. 6. Major shift in wavelength is observed in the response of biosensor with different heights of GST layer, which may be due to changes in variation of refractive index as the height changes. As discussed in the design and modeling section, the perfect absorption can be achieved by using the appropriate types of material between the two plates, with suitable physical properties. Changing the distance between two plates results in variation in resonance intensity, which leads to variation in the energy confinement between the two plates; variation in energy confinement is reflected as variation in absorption intensity.

**Figure 7:**
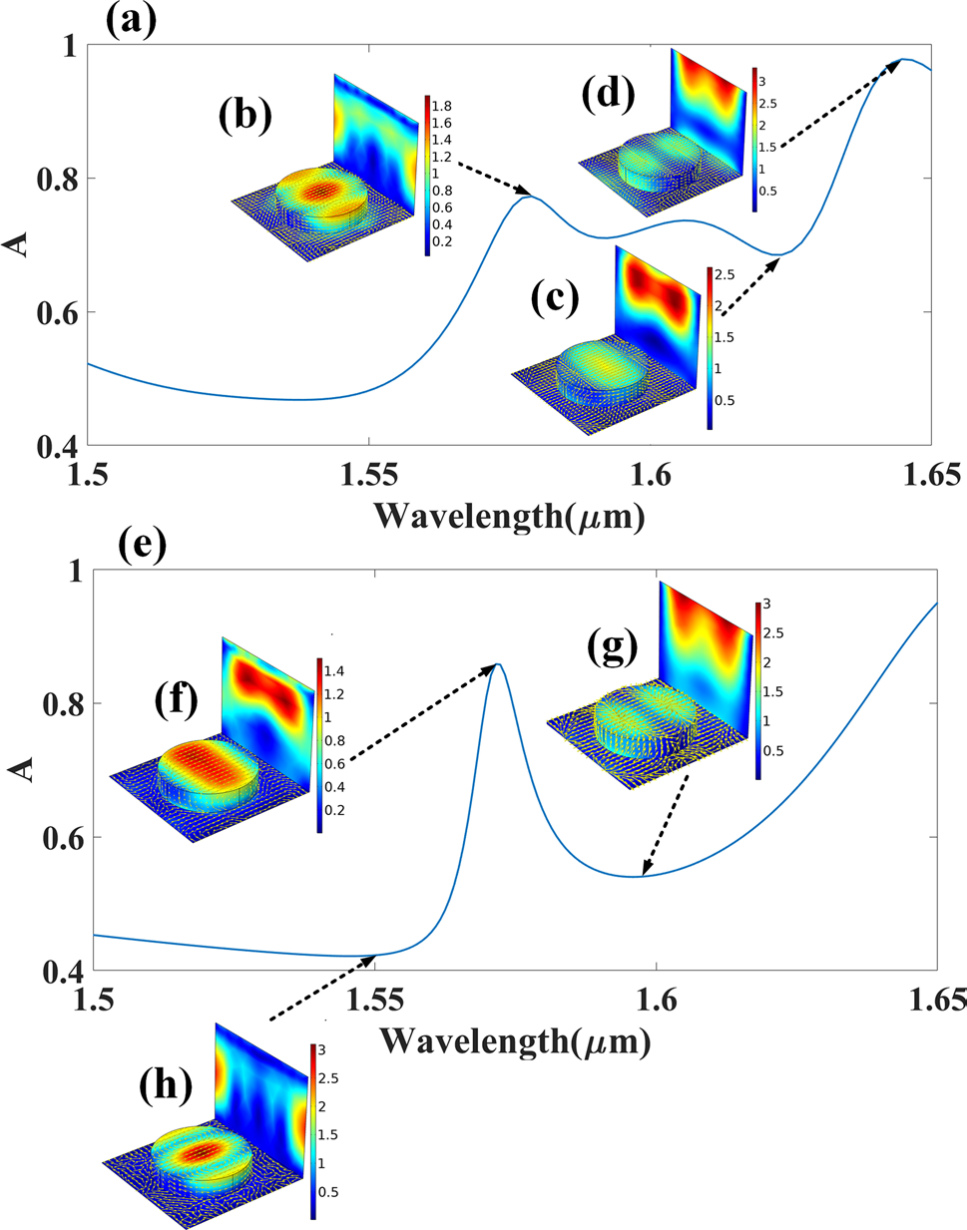
Normalized electric field intensity at different resonance points of the aGST and cGST-based biosensor with cylindrical resonator array. (**a**) Absorption spectra (1.5–1.65 µm) of the hemoglobin with refractive index of 1.336 and cGST as substrate. The normalized electric field intensity with current distribution over a surface at (**b**) 1.574 µm, (**c**) 1.67 µm, (**d**) 1.64 µm with cGST as substrate. (**e**) Absorption spectra (1.5–1.65 µm) of the hemoglobin with refractive index of 1.336 and aGST as substrate. The normalized electric field intensity with current distribution over a surface at (**f**) 1.58 µm, (**g**) 1.63 µm, (**h**) 1.64 µm with aGST as substrate.

Figure 7 shows the response of the normalized electric field intensity in aGST and cGST-built biosensor with unit cell assigned as UC1; also shown is the surface current density at every resonance point. Strong electric fields on the resonating structure can be observed at the wavelength with large absorption peaks. In some cases, the dipole moment generated at the surface of the resonators can also be observed. A strong energy confinement leads to high absorption intensity at a particular resonating point. An extensive comparative analysis of the proposed structures with the previously reported designs in terms of dimensions, operating band, sensitivity and material used is presented in this paper; Table 1 shows a large wavelength shift of the absorption peak with the use of GST. The proposed structures of biosensor have also been proven tunable as GST transitions into different phases. The structures can be fabricated using common deposition techniques^50^, combined with a well-developed laser interference lithography technique and dry etching method^51^.

**Table 1:** Comparative analysis of the proposed biosensor designs with the previously reported findings.

Work	Dimensions (W × L × H) μm^3^	Operating band (μm)	Sensitivity (nm/RIU)	Material
This work (cylindrical resonators)	UC2 × 2.8	1.5–1.65	2000	Au-GST
This work (cubical resonators)			2667	Au-GST
^52^	–	0.4–1	191	Glass-Au
^53^	0.6 × 0.6 × 0.63	0.74–5.9	273	Glass-MgF_2_
^54^	0.8 × 0.8 × 0.9	1–2.5	1518	Glass-Au
^55^	–	0.9–1.1	1100	Ag-Air
^56^	0.5 × 0.5 × 0.6	0.6–1.5	1194	Glass-Au

## Conclusion

A tunable and highly sensitive refractive index-based biosensor has been numerically investigated within the range of 1.5–1.65 µm wavelength. Different phases of GST (aGST and cGST) used in the proposed structures of biosensor has been investigated. The different structures of metamaterial (cubic and cylindrical) gold resonator and the respective wavelength shift have also been investigated. The absorption spectra of different structures and arrays have been investigated in terms variation in sensitivity. High absorption intensity can be associated with the high refractive index resulted from the use of GST in the construction of biosensor. Tunable wavelength shift can also be demonstrated with the use of analyte in different concentrations. The effects of physical parameters on the absorption intensity within the proposed range of wavelength have also been investigated. Major variation in absorption spectra can be observed in the application of aGST and cGST in biosensor. The overall bandwidth and resonating region are different for different phases of GST, i.e., a wavelength shift of 300 nm and 150 nm can be observed in detection of urine and hemoglobin, respectively. The proposed structures of biosensor demonstrate variation in sensitivity within the range of 773–2667 nm/RIU. The tunable response of the proposed structures of biosensor can be potentially improved by investigating the responsiveness toward smaller biomolecules down to molecular size.

## Supplementary Information

Supplementary Information

## References

[1] Rocha-Santos TAP (2014). Sensors and biosensors based on magnetic nanoparticles. TrAC Trends Anal. Chem..

[2] Hakimian F, Ghourchian H, Hashemi AS, Arastoo MR, Behnam Rad M (2018). Ultrasensitive optical biosensor for detection of miRNA-155 using positively charged Au nanoparticles. Sci. Rep..

[3] Baaske MD, Foreman MR, Vollmer F (2014). Single-molecule nucleic acid interactions monitored on a label-free microcavity biosensor platform. Nat. Nanotechnol..

[4] Kaushik S, Tiwari UK, Deep A, Sinha RK (2019). Two-dimensional transition metal dichalcogenides assisted biofunctionalized optical fiber SPR biosensor for efficient and rapid detection of bovine serum albumin. Sci. Rep..

[5] Sztilkovics M (2020). Single-cell adhesion force kinetics of cell populations from combined label-free optical biosensor and robotic fluidic force microscopy. Sci. Rep..

[6] Yoo SM, Lee SY (2016). Optical biosensors for the detection of pathogenic microorganisms. Trends Biotechnol..

[7] Zanchetta G, Lanfranco R, Giavazzi F, Bellini T, Buscaglia M (2017). Emerging applications of label-free optical biosensors. Nanophotonics.

[8] Li X, Soler M, Özdemir CI, Belushkin A, Yesilköy F, Altug H (2017). Plasmonic nanohole array biosensor for label-free and real-time analysis of live cell secretion. Lab Chip.

[9] Schasfoort RBM (2017). Handbook of surface plasmon resonance.

[10] Zheng D, Hu X, Lin YS, Chen CH (2020). Tunable multi-resonance of terahertz metamaterial using split-disk resonators. AIP Adv..

[11] Lerner MB (2014). Scalable production of highly sensitive nanosensors based on graphene functionalized with a designed G protein-coupled receptor. Nano Lett..

[12] Patel SK (2020). Graphene-based highly sensitive refractive index biosensors using C-shaped metasurface. IEEE Sens. J..

[13] Homola J, Yee SS, Gauglitz G (1999). Surface plasmon resonance sensors: review. Sens. Actuators B Chem..

[14] Patel SK (2020). Design of graphene metasurface based sensitive infrared biosensor. Sens. Actuators A Phys..

[15] Zhang X, Lin YS, Yang BR (2020). Tunable color switch using split-ring metamaterial. Opt. Laser Technol..

[16] Xu T, Lin YS (2020). Tunable terahertz metamaterial using an electric split-ring resonator with polarization-sensitive characteristic. Appl. Sci..

[17] Vahed H, Nadri C (2019). Sensitivity enhancement of SPR optical biosensor based on Graphene–MoS_2_ structure with nanocomposite layer. Opt. Mater. (Amst).

[18] Patel SK, Parmar J, Trivedi H, Zakaria R, Nguyen TK, Dhasarathan V (2020). Highly sensitive graphene-based refractive index biosensor using gold metasurface array. IEEE Photonics Technol. Lett..

[19] Tavousi A, Rakhshani MR, Mansouri-Birjandi MA (2018). High sensitivity label-free refractometer based biosensor applicable to glycated hemoglobin detection in human blood using all-circular photonic crystal ring resonators. Opt. Commun..

[20] Hernández R, Vallés C, Benito AM, Maser WK, Xavier Rius F, Riu J (2014). Graphene-based potentiometric biosensor for the immediate detection of living bacteria. Biosens. Bioelectron..

[21] Yang X, Lu Y, Liu B, Yao J (2017). Analysis of graphene-based photonic crystal fiber sensor using birefringence and surface plasmon resonance. Plasmonics.

[22] Zhao X (2019). Optically modulated ultra-broadband all-silicon metamaterial terahertz absorbers. ACS Photonics.

[23] Dai J, Xu R, Lin YS, Chen CH (2020). Tunable electromagnetic characteristics of suspended nanodisk metasurface. Opt. Laser Technol..

[24] Luan E, Shoman H, Ratner DM, Cheung KC, Chrostowski L (2018). Silicon photonic biosensors using label-free detection. Sensors (Switzerland).

[25] Huang Y, Xu J, Liu J, Wang X, Chen B (2017). Disease-related detection with electrochemical biosensors: a review. Sensors (Switzerland).

[26] Sharma AK (2013). Plasmonic biosensor for detection of hemoglobin concentration in human blood: design considerations. J. Appl. Phys..

[27] Vafapour Z, Ghahraloud H (2018). Semiconductor-based far-infrared biosensor by optical control of light propagation using THz metamaterial. J. Opt. Soc. Am. B.

[28] Li W, Cheng Y (2020). Dual-band tunable terahertz perfect metamaterial absorber based on strontium titanate (STO) resonator structure. Opt. Commun..

[29] Mo Y, Zhong J, Lin YS (2020). Tunable chevron-shaped infrared metamaterial. Mater. Lett..

[30] Xu Z, Lin Z, Cheng S, Lin Y-S (2019). Reconfigurable and tunable terahertz wrench-shape metamaterial performing programmable characteristic. Opt. Lett..

[31] Ou H, Lu F, Xu Z, Lin YS (2020). Terahertz metamaterial with multiple resonances for biosensing application. Nanomaterials.

[32] Yang W, Lin Y-S (2020). Tunable metamaterial filter for optical communication in the terahertz frequency range. Opt. Express.

[33] Zhan F, Lin Y-S (2020). Tunable multiresonance using complementary circular metamaterial. Opt. Lett..

[34] Liu P (2019). Actively tunable terahertz chain-link metamaterial with bidirectional polarization-dependent characteristic. Sci. Rep..

[35] Alvarez CN, Cheung R, Thompson JS (2017). Performance analysis of hybrid metal-graphene frequency reconfigurable antennas in the microwave regime. IEEE Trans. Antennas Propag..

[36] Lin Z, Xu Z, Liu P, Liang Z, Lin YS (2020). Polarization-sensitive terahertz resonator using asymmetrical F-shaped metamaterial. Opt. Laser Technol..

[37] La Spada L, Vegni L (2018). Electromagnetic nanoparticles for sensing and medical diagnostic applications. Materials (Basel)..

[38] Zou H, Cheng Y (2019). Design of a six-band terahertz metamaterial absorber for temperature sensing application. Opt. Mater. (Amst).

[39] Xu R, Lin YS (2020). Tunable infrared metamaterial emitter for gas sensing application. Nanomaterials.

[40] Lin YS, Dai J, Zeng Z, Yang BR (2020). Metasurface color filters using aluminum and lithium niobate configurations. Nanoscale Res. Lett..

[41] Zhang L (2020). Ultrasensitive skin-like wearable optical sensors based on glass micro/nanofibers. Opto-Electronic Adv..

[42] Guo Z, Yang X, Shen F, Zhou Q, Gao J, Guo K (2018). Active-tuning and polarization-independent absorber and sensor in the infrared region based on the phase change material of Ge2Sb2Te5 (GST). Sci. Rep..

[43] Wei M, Song Z, Deng Y, Liu Y, Chen Q (2019). Large-angle mid-infrared absorption switch enabled by polarization-independent GST metasurfaces. Mater. Lett..

[44] Gerislioglu B, Bakan G, Ahuja R, Adam J, Mishra YK, Ahmadivand A (2020). The role of Ge2Sb2Te5 in enhancing the performance of functional plasmonic devices. Mater. Today Phys.

[45] Song W-D, Shi L-P, Miao X-S, Chong C-T (2008). Synthesis and characteristics of a phase-change magnetic material. Adv. Mater..

[46] Hutsell SQ, Kimple RJ, Siderovski DP, Willard FS, Kimple AJ (2010). High-affinity immobilization of proteins using biotin- and GST-based coupling strategies. Methods Mol. Biol..

[47] Zhang L, Wang Y, Zhou L, Chen F (2019). Tunable perfect absorber based on gold grating including phase-changing material in visible range. Appl. Phys. A Mater. Sci. Process..

[48] Coombs JH, Jongenelis APJM, van Es-Spiekman W, Jacobs BAJ (1995). Laser-induced crystallization phenomena in GeTe-based alloys. I. Characterization of nucleation and growth. J. Appl. Phys..

[49] Landy NI, Sajuyigbe S, Mock JJ, Smith DR, Padilla WJ (2008). Perfect metamaterial absorber. Phys. Rev. Lett..

[50] Jahani S, Jacob Z (2016). All-dielectric metamaterials. Nat. Nanotechnol..

[51] Chen YG (2013). Hybrid phase-change plasmonic crystals for active tuning of lattice resonances. Opt. Express.

[52] Haes AJ, Van Duyne RP (2002). A nanoscale optical biosensor: sensitivity and selectivity of an approach based on the localized surface plasmon resonance spectroscopy of triangular silver nanoparticles. J. Am. Chem. Soc..

[53] Lari ES, Vafapour Z, Ghahraloud H (2020). Optically tunable triple–band perfect absorber for nonlinear optical liquids sensing. IEEE Sens. J..

[54] Cheng Y, Zhang H, Mao XS, Gong RZ (2018). Dual-band plasmonic perfect absorber based on all-metal nanostructure for refractive index sensing application. Mater. Lett..

[55] Zafar R, Nawaz S, Singh G, D’Alessandro A, Salim M (2018). Plasmonics-based refractive index sensor for detection of hemoglobin concentration. IEEE Sens. J..

[56] Cheng Y, Luo H, Chen F, Gong R (2019). Triple narrow-band plasmonic perfect absorber for refractive index sensing applications of optical frequency. OSA Contin..

