# Supplementary material for: Tunable infrared metamaterial-based biosensor for detection of hemoglobin and urine using phase change material
Source: Sci Rep. 2021 Mar 29;11:7101. doi: 10.1038/s41598-021-86700-6 (PMC8007597; doi:10.1038/s41598-021-86700-6)
Supplement: Supplementary file 1 — Supplementary Information [file 41598_2021_86700_MOESM1_ESM.docx]

**Tunable infrared metamaterial-based biosensor for detection of hemoglobin and urine using phase change material**

*Shobhit K. Patel^1,2^, Juveriya Parmar^3^, Vishal Sorathiya^1,4^, Truong Khang Nguyen^5,6,*^,Vigneswaran Dhasarathan^5,6*^*

*^1^Department of Electronics and Communication Engineering, Marwadi University, Gujarat, Rajkot, 360003 India*

*^2^Department of Computer Engineering, Marwadi Unversity, Gujarat, Rajkot, 360003 India*

*^3^Department of Physics, Marwadi University, Gujarat, Rajkot, India*

*^4^Department of Information and Communication Technology, Marwadi University, Gujarat, Rajkot, India*

*^5^Division of Computational Physics, Institute for Computational Science, Ton Duc Thang University, Ho Chi Minh City, Vietnam*

*^6^Faculty of Electrical and Electronics Engineering, Ton Duc Thang University, Ho Chi Minh City, Vietnam*

**Corresponding author: Division of Computational Physics, Institute for Computational Science, Ton Duc Thang University, Ho Chi Minh City, 700000, Vietnam, Tel.no. +84 28 37755024.*

*Email Id:* [*vigneswaran.d@tdtu.edu.vn*](mailto:vigneswaran.d@tdtu.edu.vn)*,* [*nguyentruongkhang@tdtu.edu.vn*](mailto:nguyentruongkhang@tdtu.edu.vn)

**SUPPLIMENTRY INFORMATION**

Figure S1:


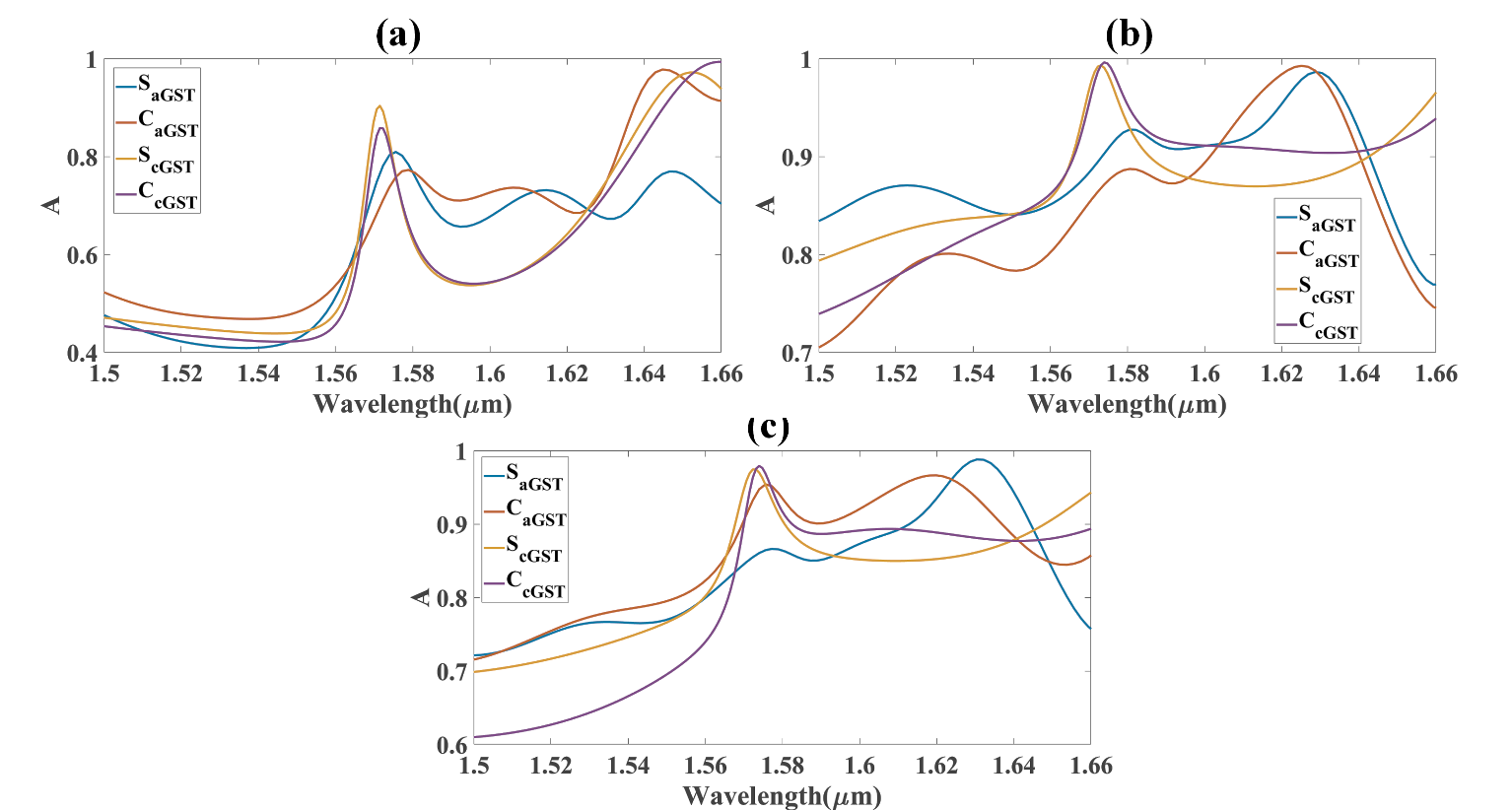


Variation in the absorption and the wavelength shift of the proposed different cylindrical and cubical array shaped based biosensor. S = Squared array and C = cubical array. Subscript representation (aGST/cGST) in each graph shows the GST material used for the response. Absorption response for (a) UC1, (b) UC2 and (c) UC3 array structure.

Comparative analysis for variation in sensitivity for the different structures is shown in Table S2 to Table S5 for different values of biosample, and GST phases. In these tables, the n_1_ - n_4_ shows the refractive indices of the urine and hemoglobin material; Δn calculated as the refractive index difference for the consecutive sample values. Similarly, Δλ calculated as the difference between the two absorption peak wavelength. Every table also showing the individual absorption peak for the different analyte samples. It can observe the maximum and minimum sensitivity variation for all the biosensor structure.

**Table S1:** Refractive index values for the biomolecules for different concentrations of samples.

| **Biomaterial-Sample** | **Hemoglobin sample concentration(g/l)** | | | | **Urine sample concentration (mg /dL)** | | | |
| --- | --- | --- | --- | --- | --- | --- | --- | --- |
| **Concentration** | 10 | 20 | 30 | 40 | 0-1.5 | 2.5 | 5 | 10 |
| **Refractive Index** | 1.3412 | 1.3607 | 1.3995 | 1.4383 | 1.336 | 1.339 | 1.342 | 1.348 |

**Table S2:** Sensitivity variation ΔS (nm/RIU) for the proposed sensor for different Urine samples for cylindric resonating structure. Table columns are shifted to show the refractive index difference (Δn), wavelength difference (Δλ) and sensitivity (S) for different biomolecules refractive indices (n1, n2, n3 and n4)

| **Array** | **Phase** | **aGST** | | | | | | **cGST** | | | | | |
| --- | --- | --- | --- | --- | --- | --- | --- | --- | --- | --- | --- | --- | --- |
|  | **n** | **n1** | **n2** | | **n3** | | **n4** | **n1** | **n2** | | **n3** | | **n4** |
|  |  | 1.336 | 1.339 | | 1.342 | | 1.348 | 1.336 | 1.339 | | 1.342 | | 1.348 |
|  | **Δn** | 0.0030 | | 0.0030 | | 0.0060 | | 0.0030 | | 0.0030 | | 0.0060 | |
| UC1 | **λ (μm)** | 1.575 | 1.58 | | 1.585 | | 1.592 | 1.571 | 1.574 | | 1.578 | | 1.585 |
|  | **Δλ (nm)** | 5 | | 5 | | 7 | | 3 | | 4 | | 7 | |
|  | **S = Δλ/Δn (nm/RIU)** | 1667 | | 1667 | | 1167 | | 1000 | | 1333 | | 1167 | |
| UC2 | **λ (μm)** | 1.58 | 1.585 | | 1.59 | | 1.599 | 1.573 | 1.576 | | 1.579 | | 1.587 |
|  | **Δλ (nm)** | 5 | | 5 | | 9 | | 3 | | 3 | | 8 | |
|  | **S = Δλ/Δn (nm/RIU)** | 1667 | | 1667 | | 1500 | | 1000 | | 1000 | | 1333 | |
| UC3 | **λ (μm)** | 1.577 | 1.583 | | 1.59 | | 1.598 | 1.573 | 1.576 | | 1.579 | | 1.586 |
|  | **Δλ (nm)** | 6 | | 7 | | 8 | | 3 | | 3 | | 7 | |
|  | **S = Δλ/Δn (nm/RIU)** | 2000 | | 2333 | | 1333 | | 1000 | | 1000 | | 1167 | |

**Table S3:** Sensitivity variation ΔS (nm/RIU) for the proposed sensor for different Haemoglobin samples for cylindric resonating structure. Table columns are shifted to show the refractive index difference (Δn), wavelength difference (Δλ) and sensitivity (S) for different biomolecules refractive indices (n1, n2, n3 and n4)

| **Array** | **Phase** | **aGST** | | | | | | **cGST** | | | | | | |
| --- | --- | --- | --- | --- | --- | --- | --- | --- | --- | --- | --- | --- | --- | --- |
|  | **n** | **n1** | **n2** | | **n3** | | **n4** | **n1** | **n2** | | **n3** | | **n4** |  |
|  |  | 1.3412 | 1.3607 | | 1.3995 | | 1.4383 | 1.3412 | 1.3607 | | 1.3995 | | 1.4383 |  |
|  | **Δn** | 0.0195 | | 0.0388 | | 0.0388 | | 0.0195 | | 0.0388 | | 0.0388 | |  |
| UC1 | **λ (μm)** | 1.582 | 1.604 | | 1.644 | | 1.712 | 1.577 | 1.6 | | 1.64 | | 1.692 |  |
|  | **Δλ (nm)** | 22 | | 40 | | 68 | | 23 | | 40 | | 52 | |  |
|  | **S = Δλ/Δn (nm/RIU)** | 1128 | | 1031 | | 1753 | | 1179 | | 1031 | | 1340 | |  |
| UC2 | **λ (μm)** | 1.587 | 1.607 | | 1.65 | | 1.701 | 1.578 | 1.601 | | 1.646 | | 1.678 |  |
|  | **Δλ (nm)** | 20 | | 43 | | 51 | | 23 | | 45 | | 32 | |  |
|  | **S = Δλ/Δn (nm/RIU)** | 1026 | | 1108 | | 1314 | | 1179 | | 1160 | | 825 | |  |
| UC3 | **λ (μm)** | 1.572 | 1.607 | | 1.659 | | 1.713 | 1.578 | 1.601 | | 1.646 | | 1.692 |  |
|  | **Δλ (nm)** | 35 | | 52 | | 54 | | 23 | | 45 | | 46 | |  |
|  | **S = Δλ/Δn (nm/RIU)** | 1795 | | 1340 | | 1392 | | 1179 | | 1160 | | 1186 | |  |

**Table S4:** Sensitivity variation ΔS (nm/RIU) for the proposed sensor for different Urine samples for metamaterial cubical resonating structure. Table columns are shifted to show the refractive index difference (Δn), wavelength difference (Δλ) and sensitivity (S) for different biomolecules refractive indices (n1, n2, n3 and n4)

| **Array** | **Phase** | **aGST** | | | | | | **cGST** | | | | | |
| --- | --- | --- | --- | --- | --- | --- | --- | --- | --- | --- | --- | --- | --- |
|  | **N** | **n1** | **n2** | | **n3** | | **n4** | **n1** | **n2** | | **n3** | | **n4** |
|  |  | 1.336 | 1.339 | | 1.342 | | 1.348 | 1.336 | 1.339 | | 1.342 | | 1.348 |
|  | **Δn** | 0.0030 | | 0.0030 | | 0.0060 | | 0.0030 | | 0.0030 | | 0.0060 | |
| UC1 | **λ (μm)** | 1.575 | 1.582 | | 1.587 | | 1.594 | 1.571 | 1.575 | | 1.578 | | 1.585 |
|  | **Δλ (nm)** | 7 | | 5 | | 7 | | 4 | | 3 | | 7 | |
|  | **S = Δλ/Δn (nm/RIU)** | 2333 | | 1667 | | 1167 | | 1333 | | 1000 | | 1167 | |
| UC2 | **λ (μm)** | 1.58 | 1.585 | | 1.59 | | 1.597 | 1.573 | 1.577 | | 1.58 | | 1.588 |
|  | **Δλ (nm)** | 5 | | 5 | | 7 | | 4 | | 3 | | 8 | |
|  | **S = Δλ/Δn (nm/RIU)** | 1667 | | 1667 | | 1167 | | 1333 | | 1000 | | 1333 | |
| UC3 | **λ (μm)** | 1.572 | 1.58 | | 1.583 | | 1.59 | 1.573 | 1.577 | | 1.581 | | 1.588 |
|  | **Δλ (nm)** | 8 | | 3 | | 7 | | 4 | | 4 | | 7 | |
|  | **S = Δλ/Δn (nm/RIU)** | 2667 | | 1000 | | 1167 | | 1333 | | 1333 | | 1167 | |

**Table S5:** Sensitivity variation ΔS (nm/RIU) for the proposed sensor for different Haemoglobin samples for metamaterial cubical resonating structure. Table columns are shifted to show the refractive index difference (Δn), wavelength difference (Δλ) and sensitivity (S) for different biomolecules refractive indices (n1, n2, n3 and n4)

| **Array** | **Phase** | **aGST** | | | | | | **cGST** | | | | | |
| --- | --- | --- | --- | --- | --- | --- | --- | --- | --- | --- | --- | --- | --- |
|  | **N** | **n1** | **n2** | | **n3** | | **n4** | **n1** | **n2** | | **n3** | | **n4** |
|  |  | 1.3412 | 1.3607 | | 1.3995 | | 1.4383 | 1.3412 | 1.3607 | | 1.3995 | | 1.4383 |
|  | **Δn** | 0.0195 | | 0.0388 | | 0.0388 | | 0.0195 | | 0.0388 | | 0.0388 | |
| UC1 | **λ (μm)** | 1.582 | 1.605 | | 1.645 | | 1.682 | 1.578 | 1.6 | | 1.64 | | 1.682 |
|  | **Δλ (nm)** | 23 | | 40 | | 37 | | 22 | | 40 | | 42 | |
|  | **S = Δλ/Δn (nm/RIU)** | 1179 | | 1031 | | 954 | | 1128 | | 1031 | | 1082 | |
| UC2 | **λ (μm)** | 1.588 | 1.611 | | 1.648 | | 1.678 | 1.579 | 1.602 | | 1.647 | | 1.692 |
|  | **Δλ (nm)** | 23 | | 37 | | 30 | | 23 | | 45 | | 45 | |
|  | **S = Δλ/Δn (nm/RIU)** | 1179 | | 954 | | 773 | | 1179 | | 1160 | | 1160 | |
| UC3 | **λ (μm)** | 1.582 | 1.604 | | 1.646 | | 1.716 | 1.579 | 1.602 | | 1.648 | | 1.69 |
|  | **Δλ (nm)** | 22 | | 42 | | 70 | | 23 | | 46 | | 42 | |
|  | **S = Δλ/Δn (nm/RIU)** | 1128 | | 1082 | | 1804 | | 1179 | | 1186 | | 1082 | |
